# Clinical, RCT and real word evidence for early intensive treatment and complex multimodal treatment of treatment resistant mental disorders

**DOI:** 10.1192/j.eurpsy.2025.108

**Published:** 2025-08-26

**Authors:** B. Baune, The PsychSTRATA Study Group and the PROMPT Study Group

**Affiliations:** Department of Psychiatry, University of Münster, Münster, Germany

## Abstract

**Abstract:**

Schizophrenia (SCZ), bipolar (BD) and major depression disorder (MDD) are severe psychiatric disorders that are challenging to treat, often leading to treatment resistance (TR). It is crucial to develop effective methods to identify and treat patients at risk of TR at an early stage in a personalized manner, considering their biological basis, their clinical and psychosocial characteristics. Effective translation of theoretical knowledge into clinical practice is essential for achieving this goal. We describe the methodology of the PROMPT project that aims at the development of a precision medicine algorithm that would help early detection of non-responder patients, who might be more prone to later develop TRD. In addition, we demonstrate other ongoing comprehensive protocols in Precision Psychiatry, such as the Horizon Europe funded PsychSTRATA consortium. To address these objectives, the PROMPT project which is organized in 2 phases will involve 300 patients with MDD already recruited in phase 1, comprising 150 TRD and 150 responders. In phase 2, a new naturalistic cohort of 300 MDD patients will be recruited to assess, under real-world conditions, the capability of the algorithm to correctly predict the treatment outcomes. Moreover, in this phase a shared decision making (SDM) process in the context of pharmacogenetic testing is explored and various needs and perspectives of different stakeholders toward the use of predictive tools for MDD treatment to foster active participation and patients’ empowerment are evaluated. In addition to this effort, the Psych-STRATA consortium addresses a major research gap in Precision Psychiatry through a seven-step approach. First, transdiagnostic biosignatures of SCZ, BD and MDD are identified by GWAS and multi-modal omics signatures associated with treatment outcome and TR (steps 1 and 2). In a next step (step 3), a randomized controlled intervention study is conducted to test the efficacy and safety of an early intensified pharmacological treatment. Following this RCT, a combined clinical and omics-based algorithm will be developed to estimate the risk for TR. This algorithm-based tool will be designed for early detection and management of TR (step 4). This algorithm will then be implemented into a framework of shared treatment decision-making with a novel mental health board (step 5). The final focus of the project is based on patient empowerment, dissemination and education (step 6) as well as the development of a software for fast, effective and individualized treatment decisions (step 7). Both the PROMPT study and the PsychSTRATA project have the potential to change the current trial and error treatment approach towards an evidence-based individualized treatment setting that takes TR risk into account at an early stage.

**Disclosure of Interest:**

None Declared

